# Carnitine Deficiency and Pregnancy

**DOI:** 10.1155/2015/101468

**Published:** 2015-05-28

**Authors:** Anouk de Bruyn, Yves Jacquemyn, Kristof Kinget, François Eyskens

**Affiliations:** ^1^Department of Obstetrics and Gynaecology, Antwerp University Hospital UZA, 2650 Edegem, Belgium; ^2^Department of Obstetrics and Gynaecology, Klina Hospital, 2930 Brasschaat, Belgium; ^3^Department of Metabolic Disorders in Children, Antwerp University Hospital UZA, 2650 Edegem, Belgium; ^4^Center of Inherited Metabolic Diseases, Metabolic Lab PCMA, 2610 Wilrijk, Belgium

## Abstract

We present two cases of carnitine deficiency in pregnancy. In our first case, systematic screening revealed L-carnitine deficiency in the first born of an asymptomatic mother. In the course of her second pregnancy, maternal carnitine levels showed a deficiency as well. In a second case, a mother known with carnitine deficiency under supplementation was followed throughout her pregnancy. Both pregnancies had an uneventful outcome. Because carnitine deficiency can have serious complications, supplementation with carnitine is advised. This supplementation should be continued throughout pregnancy according to plasma concentrations.

## 1. Introduction

Carnitine (*β*-hydroxy-y-N-trimethylammonium butyrate) is necessary for the transport of long chain fatty acids across the inner mitochondrial membrane, where they are used as substrates for the beta-oxidation cycle. Beta-oxidation is the most important source of energy in case of exercise of low and mild intensity and during fasting. Levocarnitine is the biologically active form. Endogenous production of the compound from lysine and methionine is primarily located in the liver, kidneys, and brain. Exogenous supply is via red meat and milk products [1].

Carnitine deficiency can have multiple causes. One of them is a condition called systemic primary carnitine deficiency (SPCD). SPCD (OMIM 212140) is a rare autosomal recessive disorder, caused by homozygous or compound heterozygous mutation in the SLC22A5 gene. As a consequence, there is a dysfunctional transporter, that is, the organic cation transporter novel 2. This transporter is located at the apical plasma membranes particularly in heart, muscle, and kidney and normally transfers carnitine intracellularly. The dysfunction leads to high renal loss of carnitine and lower concentrations of carnitine in blood and tissues. Thus, lower concentrations of carnitine will be available for the transfer of long chain fatty acids [2, 3]. SPCD has many variations in its presentation, from asymptomatic over metabolic crises at young age (sudden infant death syndrome, episodes of hypoglycemia, and Reye-like syndrome) to progressive cardiomyopathy [4].

Carnitine can cross the placenta. Hence a low carnitine level of the neonate can reflect both neonatal deficiency and maternal deficiency [5–7].

## 2. Case Presentation

*Case  1*. The first patient delivered a healthy term girl after an uneventful pregnancy. Blood results in the context of the Flemish neonatal screening program showed low free carnitine. Supplementation with L-carnitine was started (200 mg/day or 50 mg/kg body weight/day) for the baby. The mother did not agree to have a blood test herself and presented herself three years later, at the age of 29 years, to our prenatal unit at 7 weeks of gestational age. At that moment she agreed for a blood test. Results were as follows: free carnitine 6 *μ*mol (normal range 32–60 *μ*mol); total carnitine 7 *μ*mol (normal values 41–70 *μ*mol); acylcarnitine 1 *μ*mol (normal values 6–15 *μ*mol); acyl/total ratio 0.14 (normal range 0.12–0.30). She was started on oral L-carnitine supplementation 500 mg 3 times a day. Plasma concentrations were monitored. At 3 months of gestation, the value of free carnitine showed a severe deficiency (3.58 *μ*mol). This was due to medication nonadherence.

**Figure 1:**
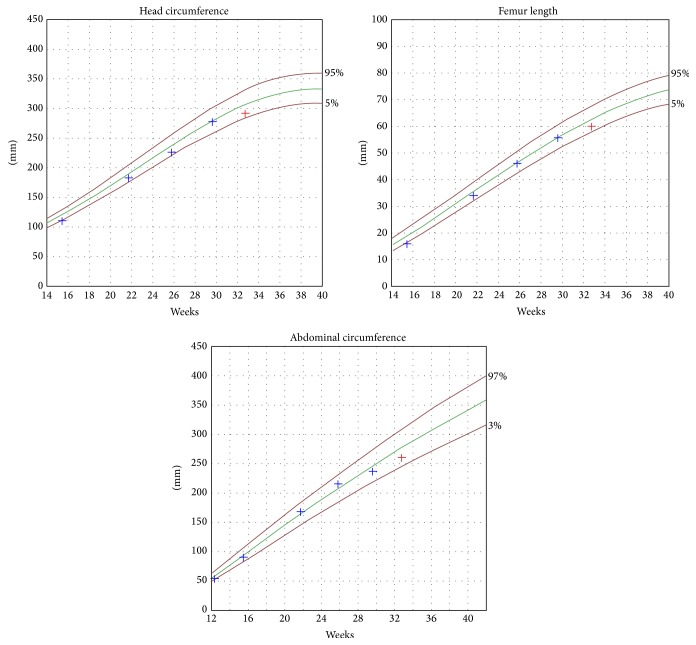
Fetal growth percentiles of case 1.

Because of the carnitine deficiency, she was considered a high-risk patient and close follow-up with ultrasound was established. Fetal growth was normal at the 25th percentile (Figure 1: fetal growth percentiles of case 1).

The course of her pregnancy was uneventful until the 39th week; at which moment she presented herself with less fetal movements and oligohydramnios on ultrasound. Labour was induced with vaginal prostaglandins and she gave birth to a son with a birth weight of 2710 grams, length of 48 cm, head circumference of 33 cm, and Apgar scores of 9, 9, and 10. Blood analysis of the umbilical cord showed normal pH values (arterial pH = 7.35 and venous pH = 7.42).

Neonatal screening of her newborn son showed low-normal carnitine levels: 7.15 *μ*mol. Supplementation was not started at that moment; the patient did not present for further follow-up.

*Case  2*. The second patient had been diagnosed with a carnitine deficiency during the investigation of a bilateral blepharoptosis and muscle pains during exercise. As she was followed in another hospital during her first pregnancy, no details could be obtained besides the performance of a caesarean section for breech presentation. She gave birth to a son that was diagnosed with a carnitine deficiency as well. At the age of 34 years, she presented at the 5th week of amenorrhea to the prenatal clinic (G2P1). Her supplementation with L-carnitine was augmented during her pregnancy from 4 to 8 g per day. Except for chronic hypertension, which was controlled with methyldopa orally, no other problems during pregnancy were reported. Close monitoring of the carnitine levels was performed and revealed (near to) normal values throughout the pregnancy, with a minimum of 20.6 *μ*mol/L and a maximal value of 32.1 *μ*mol/L.

A repeat caesarean section was performed at 38 weeks and 5 days. A healthy girl was born with a birth weight of 3606 grams, length of 50.5 cm, head circumference of 35.5 cm, and Apgar scores of 9, 9, and 10. Initial blood results of the newborn girl showed normal carnitine levels (36 *μ*mol/L). However, three months postnatally, a decreased serum carnitine (17 *μ*mol/L) was seen, so supplementation with 600 mg/d carnitine was started. After her second pregnancy, further investigations were performed on the mother. A renal excretion of L-carnitine in the urine exceeded 80% of the amount filtrated within 24 hours (normal <5%). DNA analysis revealed a heterozygotic mutation in the SLC22A5 gene and no mutation was found in the other allele, so there was no diagnosis of SPCD.

## 3. Discussion

Neonatal screening is one of the detection manners of SPCD. Among others, Lee, Schimmenti, and El-Hattab described cases where neonatal SPCD as well as maternal SPCD was detected after neonatal screening [5–7]. Primary presentation with maternal symptoms during pregnancy is another way to suspect SPCD. This is a consequence of the higher metabolism present during pregnancy. Other causes of carnitine deficiency besides mutations in the SLC22A5 gene are classified as secondary carnitine deficiency and include other hereditary metabolic diseases (e.g., fatty acid oxidation defects), medication (valproic acid, cyclosporine, and pivampicillin), malnutrition, hemodialysis and renal tubular dysfunction (Fanconi nephropathy), and prematurity (lower placentary transfer). Lower carnitine levels are also seen in vegetarians (20–30% lower concentrations).

To differentiate SPCD and other mutations in the SLC22A5 gene (as seen in case 2) from alternative causes of carnitine deficiency, a thorough anamnesis and supplementary tests are suggested. Blood analysis will reveal low carnitine plasma concentrations, with a normal acyl/total carnitine ratio. A 24-hour urinary excretion of L-carnitine under L-carnitine supplementation will typically reveal very high rates of carnitine excretion in SPCD, but without the presence of abnormal organic acids as seen in secondary carnitine deficiency.

The final diagnosis can be made through genetic analysis (to prove the mutation(s)) or through measurement of the carnitine transport in fibroblasts, isolated from a skin biopsy. This will be reduced in case of SPCD (<10% of controls) [1].

Little is known about the treatment of asymptomatic carnitine deficient patients [8]. Since we know that disorders in fatty acid oxidation can lead to sudden death, current recommendations are to treat also the asymptomatic patients by supplementation with L-carnitine [9]. This should be done to prevent potential decompensation with serious consequences. Drug information about carnitine proposes target plasma concentrations between 24 and 48 *μ*mol/L.

Schoderbeck et al. [10] discovered a significant decrease in the plasma concentrations of total and free carnitine during pregnancy in healthy women. Because fetal and maternal carnitine concentrations are correlated, it is important for the neonate as well that sufficient maternal carnitine concentrations be important. In patients who receive supplementation, as in patients with known SPCD, close follow-up is therefore recommended to adjust supplementation when necessary. Besides the course of a pregnancy, concentrations of carnitine in people known with a deficiency need to be checked annually.

To our knowledge, no studies have been performed to determine the ideal dose of carnitine supplementation and the target values in pregnancy.

## Learning Points/Take Home Messages

- Systematic neonatal screening can lead to diagnosis of metabolic disorders in the mother.
- Carnitine supplementation should be continued during every (following) pregnancy (according to plasma concentrations).
- Every case of SPCD should be reported to enlarge our knowledge about this pathology.
